# Reply to "comment on: Multifocal papillary thyroid microcarcinomas: is the total tumor diameter associated with the tumor behavior? A retrospective cohort study"

**DOI:** 10.31744/einstein_journal/2026CE2284

**Published:** 2026-01-28

**Authors:** Larissa Ariane de Nardi, Ana Flávia Aguiar Ribeiro Coutinho, Carlos Segundo Paiva Soares, Simone Antunes Terra, Cristiano Claudino de Oliveira, Katia Hiromoto Koga, Sonia Marta Moriguchi, José Vicente Tagliarini, Gláucia Maria Ferreira da Silva Mazeto

**Affiliations:** 1 Universidade Estadual Paulista "Júlio de Mesquita Filho" Faculdade de Medicina de Botucatu Internal Medicine Department Botucatu SP Brazil Internal Medicine Department, Faculdade de Medicina de Botucatu, Universidade Estadual Paulista "Júlio de Mesquita Filho", Botucatu, SP, Brazil.; 2 Universidade Estadual Paulista "Júlio de Mesquita Filho" Faculdade de Medicina de Botucatu Otorhinolaryngology and Head and Neck Surgery Department Botucatu SP Brazil Ophthalmology, Otorhinolaryngology and Head and Neck Surgery Department, Faculdade de Medicina de Botucatu, Universidade Estadual Paulista "Júlio de Mesquita Filho", Botucatu, SP, Brazil.; 3 Universidade Estadual Paulista "Júlio de Mesquita Filho" Faculdade de Medicina de Botucatu Pathology Department Botucatu SP Brazil Pathology Department, Faculdade de Medicina de Botucatu, Universidade Estadual Paulista "Júlio de Mesquita Filho", Botucatu, SP, Brazil.; 4 A.C.Camargo Cancer Center Department of Pathological Anatomy São Paulo SP Brazil Department of Pathological Anatomy, A.C.Camargo Cancer Center, São Paulo, SP, Brazil.; 5 Universidade Estadual Paulista "Júlio de Mesquita Filho" Faculdade de Medicina de Botucatu Nuclear Medicine Department Botucatu SP Brazil Nuclear Medicine Department, Faculdade de Medicina de Botucatu, Universidade Estadual Paulista "Júlio de Mesquita Filho", Botucatu, SP, Brazil.

Dear Editor

We are deeply grateful to Dr. Ilker Sengul and Dr. Demet Sengul for their careful consideration of our article.^(1)^ We feel honored by their interest in our work, particularly given their recognized expertise in this field. Below we present our considerations regarding the comments they kindly provided.

Indeed, the use of total tumor diameter (TTD) as a potential marker of tumor behavior in multifocal papillary thyroid microcarcinoma (PTMC) has been a subject of ongoing debate in the literature. Studies using different methodologies and patient populations have reported variable findings regarding its prognostic significance.^(2,3)^

In our aforementioned study there was no association between TTD—or other parameters considered markers of tumor behavior—and the proposed outcomes.^(1)^ Several factors may account for this, including the rigorous selection of our sample in accordance with the inclusion criteria, as well as certain methodological limitations which are discussed in a specific paragraph of the original article.^(1)^ Therefore, we agree that studies with more robust designs and larger sample sizes are needed to further investigate this issue, and to more precisely define the role of TTD in the management of multifocal PTMC. We also agree that the observed association between the number of metastatic lymph nodes and greater initial aggressiveness is very interesting.^(1)^ Because this parameter has previously been associated with recurrence-free survival in PTMC,^(4)^ its use in the risk assessment of relevant cases should indeed be considered.

We would like to conclude by once again thanking Dr. Ilker Sengul and Dr. Demet Sengul for their constructive comments, which have contributed to advancing discussion on this important topic.

## Data Availability

After publication, data will be available from the authors upon request—this condition is justified in the manuscript.
